# Interstitial lung abnormalities and interstitial lung diseases associated with cigarette smoking in a rural cohort undergoing surgical resection

**DOI:** 10.1186/s12890-022-01961-9

**Published:** 2022-04-29

**Authors:** Rahul G. Sangani, Vishal Deepak, Andrew J. Ghio, Michael J. Forte, Rafia Zulfikar, Zalak Patel, Austin King, Esra Alshaikhnassir, Ghulam Abbas, Jeffrey Vos

**Affiliations:** 1 Section of Pulmonary, Critical Care and Sleep Medicine, Department of Medicine, West Virginia University School of Medicine, West Virginia University, 1 Medical Center Dr., PO Box 9166, Morgantown, WV 26506 USA; 2 Human Studies Facility, US EPA, Chapel Hill, NC USA; 3 Department of Radiology, West Virginia University, Morgantown, WV USA; 4 Section of Internal Medicine, Department of Medicine, West Virginia University, Morgantown, WV USA; 5 Deparment of Pathology, West Virginia University, Morgantown, WV USA; 6 Deparment of Cardiovascular and Thoracic Surgery, West Virginia University, Morgantown, WV USA

**Keywords:** Interstitial lung abnormalities, Interstitial lung diseases, Emphysema, Cigarette smoking, Fibrosis, Histopathology and peribronchiolar metaplasia

## Abstract

**Background:**

Cigarette smoking is a risk factor for interstitial lung abnormalities (ILAs) and interstitial lung diseases (ILDs). Investigation defining the relationships between ILAs/ILDs and clinical, radiographic, and pathologic findings in smokers have been incomplete. Employing a cohort undergoing surgical resection for lung nodules/masses, we (1) define the prevalence of ILAs/ILDs, (2) delineate their clinical, radiographic and pathologic predictors, and (3) determine their associations with mortality.

**Methods:**

Patients undergoing resection of lung nodules/masses between 2017 and 2020 at a rural Appalachian, tertiary medical center were retrospectively investigated. Predictors for ILAs/ILDs and mortality were assessed using multivariate logistic regression analysis.

**Results:**

In the total study cohort of 352 patients, radiographic ILAs and ILDs were observed in 35.2% and 17.6%, respectively. Among ILA patterns, subpleural reticular changes (14.8%), non-emphysematous cysts, centrilobular (CL) ground glass opacities (GGOs) (8% each), and mixed CL-GGO and subpleural reticular changes (7.4%) were common. ILD patterns included combined pulmonary fibrosis emphysema (CPFE) (3.1%), respiratory bronchiolitis (RB)-ILD (3.1%), organizing pneumonitis (2.8%) and unclassifiable (4.8%). The group with radiographic ILAs/ILDs had a significantly higher proportion of ever smokers (49% vs. 39.9%), pack years of smoking (44.57 ± 36.21 vs. 34.96 ± 26.22), clinical comorbidities of COPD (35% vs. 26.5%) and mildly reduced diffusion capacity (% predicated 66.29 ± 20.55 vs. 71.84 ± 23). Radiographic centrilobular and paraseptal emphysema (40% vs. 22.2% and 17.6% vs. 9.6%, respectively) and isolated traction bronchiectasis (10.2% vs. 4.2%) were associated with ILAs/ILDs. Pathological variables of emphysema (34.9% vs. 18.5%), any fibrosis (15.9% vs. 4.6%), peribronchiolar metaplasia (PBM, 8% vs. 1.1%), RB (10.3% vs. 2.5%), and anthracosis (21.6% vs. 14.5%) were associated with ILAs/ILDs. Histologic emphysema showed positive correlations with any fibrosis, RB, anthracosis and ≥ 30 pack year of smoking. The group with ILAs/ILDs had significantly higher mortality (9.1% vs. 2.2%, OR 4.13, [95% CI of 1.84–9.25]).

**Conclusions:**

In a rural cohort undergoing surgical resection, radiographic subclinical ILAs/ILDs patterns were highly prevalent and associated with ever smoking and intensity of smoking. The presence of radiographic ILA/ILD patterns and isolated honeycomb changes were associated with increased mortality. Subclinical ILAs/ILDs and histologic fibrosis correlated with clinical COPD as well as radiographic and pathologic emphysema emphasizing the co-existence of these pulmonary injuries in a heavily smoking population.

**Supplementary Information:**

The online version contains supplementary material available at 10.1186/s12890-022-01961-9.

## Introduction

Cigarette smoking has been associated with the precursor lesions of interstitial lung abnormalities (ILAs) and a diversity of interstitial lung diseases (ILDs) including respiratory bronchiolitis–interstitial lung disease (RB-ILD), desquamative interstitial pneumonia (DIP), pulmonary Langerhans cell histiocytosis (PLCH), idiopathic pulmonary fibrosis (IPF), and combined pulmonary fibrosis and emphysema (CPFE) [1]. Penetration of smoking-related pulmonary injuries is variable with a prevalence of 3 to 17% (mean of 8%) for ILAs, < 1% for CPFE and 0.0005–0.002% for IPF [2]. Except for subclinical ILAs, these diseases often present with progressive dyspnea and some combination of other symptoms (cough, sputum and wheezing). Attempts to identify an individual lung disease overlooks co-existing pathology and disease following exposure to the shared risk factor of cigarette smoking [3, 4]. Investigation of the associations between subclinical ILAs and other cigarette smoking-related lung diseases have sometimes provided discrepant results. While identifying the potential role of current smoking in ILAs, Washko et al. demonstrated an inverse relationship with emphysema, with prevalence of 8% in COPD gene cohort [5]. These findings may have been confounded by the exclusion of radiographic emphysematous regions which may include smoking-related interstitial fibrosis, lack of histologic confirmation, and approximately one-third of patients meeting criteria for “indeterminate” ILA [6, 7]. Although comparison of quantitative methods of ILA evaluation to visual ILA showed potential modification from coexisting emphysema (5% or more) [8], a significant positive association was demonstrated between paraseptal emphysema and ILAs [9]. Recent studies have suggested that ILAs can show a higher prevalence (23.1 to 40.7%) and are associated with both a worse clinical course and higher mortality rates in COPD patients [10–13].

The Appalachian region is challenged with the highest rates of smoking (25.2%) in the United States [14]. Subsequently, there is an excessive burden of lung disease associated with smoking in this region [15, 16]. Employing a cohort diagnosed with lung nodule/mass and undergoing surgical resection, we (1) define the prevalence of ILAs/ILDs, (2) delineate clinical, radiographic and pathologic predictors of ILAs/ILDs, and (3) determine their associations with mortality.

## Materials and methods

### Study design

**Fig. 1:**
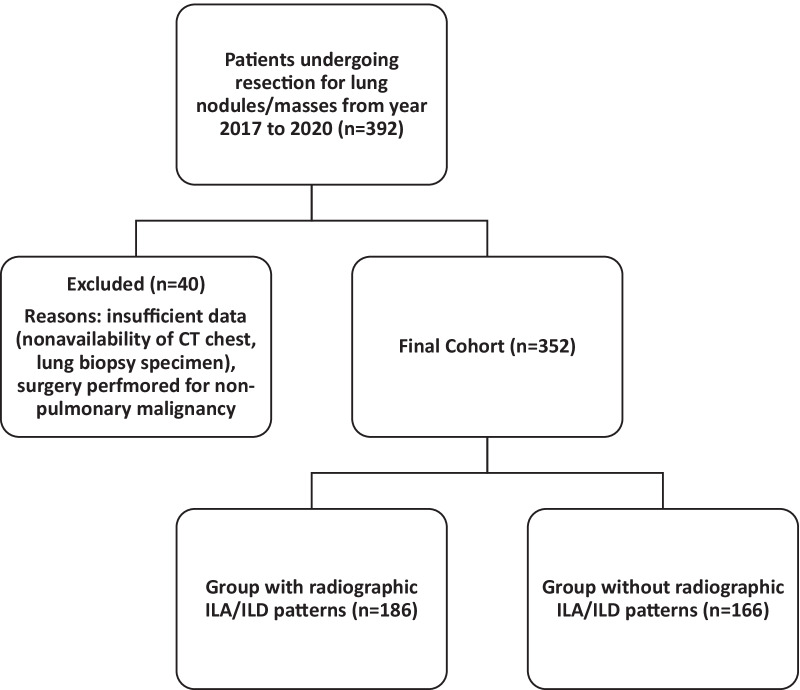
Study approach

A single-center, retrospective, cohort study was conducted at West Virginia University Hospital (WVUH). The study protocol was reviewed and approved by the institutional review board (ID# 2010131995). During the study period (January 1, 2017 to December 31, 2020), patients who underwent surgical resection for suspicious lung nodules/masses coordinated through the thoracic oncology clinic of WVUH were identified. Groups with or without radiographic ILA and ILD patterns were defined (Fig. 1). Aims of this retrospective review were to define (1) the prevalence of subclinical radiographic ILA and ILD in the cohort, (2) clinical, radiographic and pathologic predictors of ILA/ILD, and (3) the impact of ILA/ILD on mortality with its predictors. Additionally, we explored the specific histologic associations for ILA/ILD patterns, and the relationship between cigarette smoking and histologic findings including emphysema. Patients were excluded from the study cohort if (1) a good quality CT scan of the chest was not obtained six months (or less) prior to surgery, (2) adequate lung tissue was not available to allow independent review, and (3) surgery performed for non-pulmonary malignancy/metastatic disease.

### Data collection

Electronic medical charts were reviewed, and data collected including demographics, smoking status, pack-years of smoking, comorbidities, baseline supplemental oxygen use, and pulmonary function tests (PFTs).

### Radiographic evaluation

Pre-operative CT scans (1 mm slice thickness for lung windows) were analyzed by three investigators to detect ILA/ILD patterns. Consensus of radiographic findings were recorded in accordance with the case definitions [5, 17] (Additional file 1). ILAs included diffuse centrilobular (CL) GGO, subpleural reticular changes, architectural distortion, traction bronchiectasis, honeycombing, and non-emphysematous cysts involving at least 5% of a lung zone. Imaging findings were excluded if they were restricted to dependent lung zones, focal paraspinal fibrosis, focal or unilateral abnormality, interstitial pulmonary edema or aspiration related findings of tree-in-bud or patchy ground glass and definite ILD patterns. Radiographic ILD patterns were reported including usual interstitial pneumonia (UIP), probable UIP, non-specific interstitial pneumonia (NSIP), RB-ILD, PLCH, DIP, CPFE, organizing pneumonia and unclassifiable patterns [18–20].

### Pathologic evaluation

At least one tissue section, and typically 3–6 sections, were examined for interstitial lung changes. Areas distant from the tumor were evaluated. Microscopic analysis focused on identifying a variety of histopathologic patterns and features indicative of ILD [21]. Retrospective histopathologic review of 352 lung specimens was performed independently by two pathologists. Consensus of pathologic findings was obtained for all specimens.

### Statistical analysis

Descriptive statistics included means, medians, and standard deviations to summarize continuous variables and frequency distributions to describe categorical variables. Chi-square or Fisher exact tests were used to detect differences in categorical variables between the groups, while means of continuous variables were compared using two-sided independent-samples t-tests. The Mann–Whitney U test was used when normality could not be assumed. Logistic regression analysis was used to determine significant predictors of ILAs/ILDs including histological features, association of smoking and various pathologic finding and to predict mortality at the end of the study. Level of significance, α = 0.05 was used for all analyses. All analyses were conducted using statistical software SPSS version 26.0.

## Results

There was a total of 392 patients who underwent surgical resection for lung nodules/masses (Fig. 1). After exclusion of those patients without a good quality CT scan of the chest or lung tissue (n = 40, 10.2%), the final number of patients was 352 patients (89.8%) (Fig. 1). This cohort was almost entirely Caucasian (96.3%) with approximately equal numbers among the genders (57.1% female). The mean age was 66.15 ± 10.19 years and the mean body mass index (BMI) was 28.35 ± 6.88 kg/m^2^ reflecting an overweight cohort.

The group with radiographic ILA and ILD patterns (n = 186, 52.8%) included 124 (35.2%) and 62 (17.6%) patients, respectively. Findings of ILA included subpleural reticular changes (14.8%), CL-GGO, non-emphysematous cysts (8% each) and mixed CL-GGO with subpleural reticular changes (7.4%) (Table 1). ILD patterns recognized on the CT scan of the chest included UIP (0.6%), probable UIP (0.6%), NSIP (0.9%), RB-ILD (3.1%), LCH (0.6%), DIP (1.1%), CPFE (3.1%), OP (2.8%) and unclassifiable (4.8%) (Table 1). There were no significant differences between the two groups in mean age, gender, and BMI (Table 2). The group with ILA/ILD had a greater proportion of ever smokers (49% vs. 39.9%, *p* = 0.010), and pack years of exposure (44.57 ± 36.21 vs. 34.96 ± 26.22, *p* = 0.005). The total study cohort showed a high burden of comorbidities with hypertension (69.9%), hyperlipidemia (59.3%), gastro-esophageal reflux disease (GERD, 40.6%), coronary artery disease (37.2%), anxiety (32.7%), diabetes (24.4%), and hypothyroidism (18.1%) with no differences between the groups. Those patients with ILA/ILD had an increased prevalence of diagnosed COPD (35% vs. 26.4%, *p* = 0.044). Supplemental oxygen was used by 14.5% of patients in the cohort and there were no differences between the groups in this. This use of supplemental O_2_ was predominantly for COPD with only a small minority of patients in the total cohort diagnosed to have an ILD prior to surgery (1.5%).

**Table 1:** Distribution of radiographic ILA and ILD patterns in the cohort (N = 352)

Variables	N (%)
1. ILA (any)	124 (35.2)
a. Centrilobular GGO	28 (8.0)
b. Subpleural reticular changes	52 (14.8)
c. Mixed a + b	26 (7.4)
d. Non-emphysematous cysts	28 (8.0)
e. Definite ILD	62 (17.6)
f. Combination other than c	14 (4.0)
2. ILD patterns (any)	62 (17.6)
a. UIP	2 (0.6)
b. Probable UIP	2 (0.6)
c. NSIP	3 (0.9)
d. RB-ILD	11 (3.1)
e. LCH	2 (0.6)
f. DIP	4 (1.1)
g. CPFE	11 (3.1)
h. OP	10 (2.8)
i. Unclassifiable	17 (4.8)

*CPFE* combined pulmonary fibrosis and emphysema, *DIP* desquamative interstitial pneumonia, *GGO* ground glass opacity, *ILA* interstitial lung abnormalities, *ILD* interstitial lung disease, *LCH* Langerhans cell histiocytosis, *NSIP* non-specific interstitial pneumonia, *OP* organizing pneumonia, *RB-ILD* respiratory bronchiolitis–interstitial lung disease, *UIP* usual interstitial pneumonia

**Table 2:** Characteristics of groups of patients with and without radiographic ILA/ILD patterns in the cohort (N = 352)

Variables, values, n (%) or mean ± SD	Group with ILA-ILD (N = 186, 52.8%)	No ILA-ILD (N = 166, 47.2%)	Total cohort (N = 352)	*p* value
Age	66.94 ± 9.35	65.28 ± 11.02	66.15 ± 10.19	0.141
Gender (male)	81 (22.8)	70 (19.9)	151 (42.9)	0.838
Body mass index (BMI, kg/m^2^)	28.19 ± 6.68	28.54 ± 7.1	28.35 ± 6.88	0.660
Race (White)	181 (51.3)	158 (45)	339 (96.3)	0.294
Smoking status:				
1. Ever-smoker	173 (49)	140 (39.9)	313 (88.9)	***0.010***
2. Pack years	44.57 ± 34.96	34.96 ± 26.22	43.23 ± 31.86	***0.005***
Comorbidities				
1. COPD	123 (35)	93 (26.4)	216 (61.4)	***0.044***
2. ILD	3 (0.9)	2 (0.6)	5 (1.5)	0.742
3. Hypertension	134 (38.1)	112 (31.8)	246 (69.9)	0.367
4. Hyperlipidemia	111 (31.5)	98 (27.8)	209 (59.3)	0.854
5. CAD	77 (21.9)	54 (15.3)	131 (37.2)	0.078
6. CHF	18 (5.1)	11 (3.1)	29 (8.2)	0.291
7. CVA	21 (5.9)	21 (5.9)	42 (11.8)	0.708
8. PAD	29 (8.3)	16 (4.5)	45 (12.8)	0.091
9. DM	45 (12.8)	41 (11.6)	86 (24.4)	0.935
10. Atrial fibrillation	31 (8.8)	19 (5.4)	50 (14.2)	0.155
11. VTE	24 (6.8)	24 (6.8)	48 (13.6)	0.686
12. CKD	23 (6.5)	12 (3.4)	35 (9.9)	0.104
13. OSA	26 (7.4)	20 (5.7)	46 (13.1)	0.578
14. GERD	80 (22.7)	63 (17.9)	143 (40.6)	0.365
15. Anxiety	65 (18.5)	50 (14.2)	115 (32.7)	0.371
16. Hypothyroidism	31 (8.8)	33 (9.3)	64 (18.1)	0.449
17. Chronic liver dysfunction	8 (2.2)	7 (2)	15 (4.2)	0.960
18. Co-existing cancer	19 (5.4)	10 (2.8)	29 (8.2)	0.201
Home O_2_ use	29 (8.3)	22 (6.2)	51 (14.5)	0.520
PFT performed:	175 (49.7)	161 (45.7)	336 (95.4)	0.191
1. FEV_1_, % predicted	76.03 ± 18.97	76.68 ± 24.45	76.34 ± 21.76	0.781
2. FVC, % predicted	85.77 ± 17.33	85.94 ± 19.04	85.85 ± 18.16	0.932
3. Ratio FEV_1_/FVC	67.91 ± 11.09	67.57 ± 13.26	67.74 ± 12.17	0.796
4. TLC, % predicted	106.42 ± 17.60	107.24 ± 22.49	106.83 ± 20.15	0.723
5. RV, % predicted	138.53 ± 40.82	143.71 ± 22.49	141.13 ± 51.41	0.384
6. DL_CO_, % predicted	66.29 ± 20.55	71.84 ± 23.01	69.06 ± 21.95	***0.023***
Mortality (dead)	32 (9.1)	8 (2.2)	40 (11.3)	**< *0.001***

Bold italics* p*-value suggest statistical significant* p*-value (alpha < 0.05)

*CAD* coronary artery disease, *CHF* congestive heart failure, *CKD* chronic kidney disease, *COPD* chronic obstructive lung disease, *CVA* cerebrovascular accident, *DL*_*CO*_ diffusion capacity for carbon monoxide, *DM* diabetes mellitus, *FEV*_*1*_ forced expiratory volume in one second, *FVC* forced vital capacity, *GERD* gastro-esophageal reflux disease, *ILA* interstitial lung abnormalities, *ILD* interstitial lung disease, *OSA* obstructive sleep apnea, *PAD* peripheral arterial disease, *RV* residual volume, *TLC* total lung capacity, *VTE* venous thromboembolism

A majority of the study cohort (95.4%) had pre-surgical PFTs performed. Mean ratio of FEV_1_/FVC for the cohort was obstructive (67.74 ± 12.17) with evidence of air trapping (mean % predicted residual volume (RV) of 141.13 ± 51.41) and mildly reduced % predicted diffusion capacity for carbon monoxide (DL_CO_, 69.06 ± 21.95), correlating with predominant clinical diagnosis of COPD in two-third of patients. There was no significant difference between the groups with and without ILA/ILD for percent predicted forced expiratory volume in one second (FEV_1_), forced vital capacity (FVC), ratio FEV_1_/FVC, total lung capacity (TLC) and RV values. The group with ILA/ILD had a significantly reduced percent predicted DL_CO_ (66.29 ± 20.55 vs. 71.84 ± 23. *p* = 0.023) (Table 2).

Additional CT chest findings of any emphysema (44.6% vs. 25.8%, *p* < 0.001), CL emphysema (40.0% vs. 22.2%, *p* < 0.001), paraseptal emphysema (17.6% vs. 9.6%, *p* = 0.006), combination patterns of emphysema (18.2% vs. 10.2%, *p* = 0.007) and isolated traction bronchiectasis (10.2% vs. 4.3%, *p* = 0.005) were more frequent in the group with ILA/ILD patterns compared to group without. Only a minority of patients showed isolated honeycomb changes (2.2%) and there was no significant difference between the groups (Table 3).

**Table 3:** Radiographic and histopathological features of groups with and without radiographic ILA/ILD patterns in the cohort (N = 352)

Variables, values, n (%) or mean ± SD	Group with ILA-ILD (N = 186, 52.8%)	No ILA-ILD (N = 166, 47.2%)	Total cohort (N = 352)	*p* value
CT chest findings:				
1. Emphysema (any)	157 (44.6)	91 (25.8)	248 (70.4)	**< *0.001***
a. Centrilobular emphysema	141 (40.0)	78 (22.2)	219 (62.2)	**< *0.001***
b. Paraseptal emphysema	62 (17.6)	34 (9.6)	96 (27.2)	***0.006***
c. Bullous emphysema	12 (3.4)	11 (3.1)	23 (6.5)	0.957
d. Panacinar emphysema	17 (4.8)	16 (4.6)	33 (9.4)	0.885
e. Combination patterns	64 (18.2)	36 (10.2)	100 (28.4)	***0.007***
2. Isolated traction bronchiectasis	36 (10.2)	15 (4.3)	51 (14.5)	***0.005***
3. Isolated honeycombing	4 (1.1)	4 (1.1)	8 (2.2)	0.876
4. Pleural plaques	15 (4.2)	8 (2.2)	23 (6.5)	0.288
Pathological findings:				
1. Primary lung cancer pathology in the resected nodule	175 (49.8)	148 (42.2)	323 (92)	0.060
a. Adenocarcinoma	95 (27.0)	81 (23.0)	176 (50.0)	0.669
b. Squamous cell	56 (15.9)	40 (11.4)	96 (27.3)	0.206
c. Large cell	8 (2.2)	3 (0.9)	11 (3.1)	0.227
d. Small cell/neuroendocrine	12 (3.4)	21 (5.9)	33 (9.3)	0.065
e. Mixed	3 (0.9)	0	3 (0.9)	0.250
2. Emphysema	123 (34.9)	65 (18.5)	188 (53.4)	**< *0.001***
3. Any fibrosis^++^	56 (15.9)	16 (4.6)	72 (20.5)	**< *0.001***
4. Peribronchiolar metaplasia	28 (8.0)	4 (1.1)	32 (9.1)	**< *0.001***
5. RB	36 (10.3)	9 (2.5)	45 (12.8)	**< *0.001***
6. DIP	10 (2.8)	3 (0.9)	13 (3.7)	0.074
7. Cellular interstitial pneumonia	3 (0.9)	0	3 (0.9)	0.250
8. OP	14 (4.0)	7 (2.0)	21 (6.0)	0.186
9. Anthracosis	77 (21.9)	51 (14.4)	128 (36.3)	***0.043***
10. Granuloma-necrotizing	3 (0.9)	9 (2.5)	12 (3.4)	0.063
11. Granuloma-non-necrotizing, sarcoid like	7 (2)	5 (1.4)	12 (3.4)	0.691
12. Granuloma-loosely formed, HP like	2 (0.6)	0	2 (0.6)	0.500
13. Granuloma-calcified	6 (1.7)	4 (1.1)	10 (2.8)	0.639
14. Miscellaneous*	20 (5.7)	9 (2.5)	29 (8.2)	0.072
a. Chronic inflammation	4 (1.1)	0		
b. Silicotic nodule	0	3 (0.9)		
c. Consolidation/necrosis	4 (1.1)	1 (0.3)		
d. Metaplastic bone/calcification	3 (0.9)	1 (0.3)		
e. Pleural plaque	2 (0.6)	2 (0.6)		

Bold italics* p*-value suggest statistical significant* p*-value (alpha < 0.05)

*ILA* interstitial lung abnormalities, *ILD* interstitial lung disease

^++^Any fibrosis included fibrosis, fibroblastic foci, honeycombing, subpleural fibrosis, architectural distortion, and radiation fibrosis

*Miscellaneous pathologic findings in ILA/ILD group included: lymphocytic interstitial pneumonia (n = 2), foreign body giant cell reactions (n = 2), DIPNECH (n = 1), follicular bronchiolitis (n = 1), adenomatous hyperplasia (n = 1), vascular medial hypertrophy (n = 1), and bronchiectasis (n = 1). On the contrary, only additional other finding in non ILA/ILD group included carcinoid (n = 1)

Histologically, 92% of resected nodules/masses demonstrated malignancy and the group with ILA/ILD trended towards a greater prevalence of lung cancer (49.8% vs. 42.2%, *p* = 0.06). Adenocarcinoma was the most common subtype (50.0%), followed by squamous cell (27.3%) and small cell/neuroendocrine malignancy (9.4%), without significant difference between the groups. Concurrent pathological findings in the ILA/ILD group included emphysema (34.9% vs. 18.5%, *p* < 0.001), any pulmonary fibrosis (15.9% vs. 4.6%, *p* < 0.001), peribronchiolar metaplasia (PBM, 8.0% vs. 1.1%, *p* < 0.001), RB (10.3% vs. 2.5%, *p* < 0.001) and anthracosis (21.9% vs. 14.4%, *p* = 0.043) (Table 3). While pathological evidence of DIP trended towards significance in the ILA/ILD group (2.8% vs. 0.9%, *p* = 0.074), necrotizing granulomatous inflammation trended towards group without ILA/ILD (0.9% vs. 2.5%, *p* = 0.063).

**Fig. 2:**
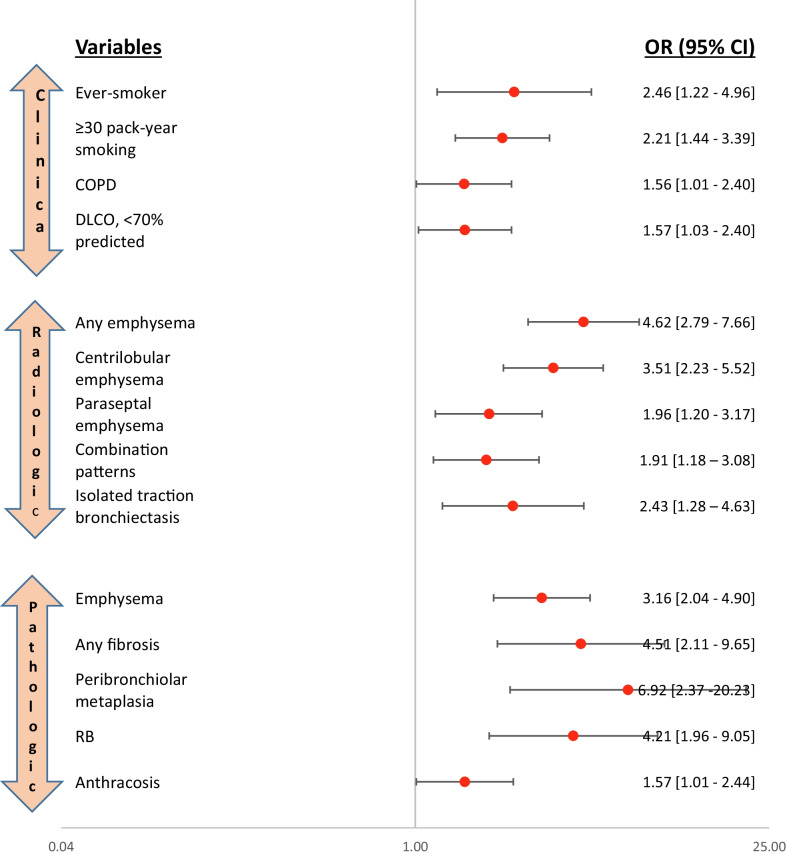
Forest plots of clinical, radiographic and pathological predictors of ILA/ILD in the cohort. *COPD* chronic obstructive lung disease, *DL*_*CO*_ diffusion capacity for carbon monoxide, *ILA* interstitial lung abnormalities, *ILD* interstitial lung disease, *RB* respiratory bronchiolitis

**Fig. 3:**
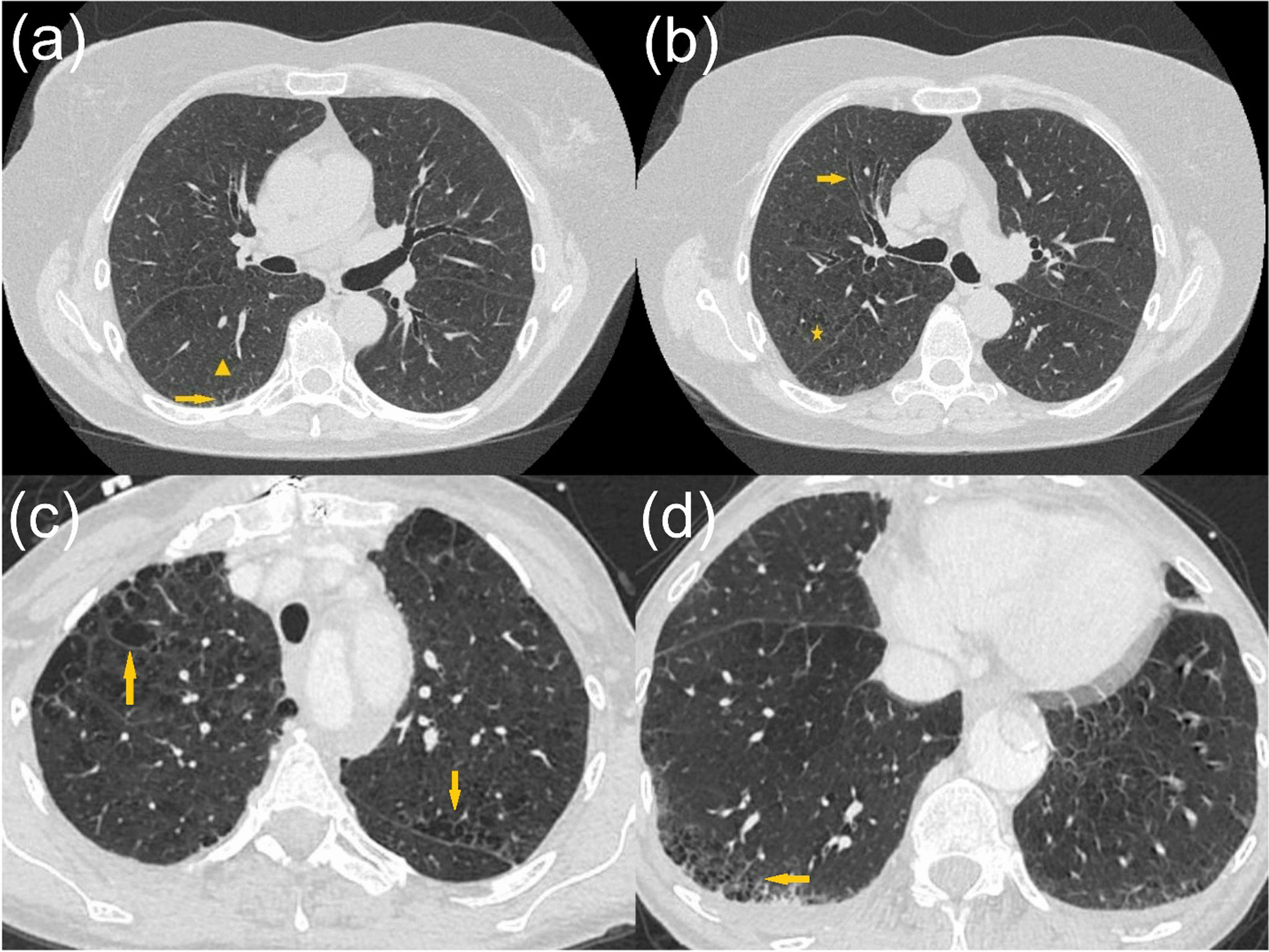
Pre-operative CT chest images of 71-year-old active smoker (55 pack years) who underwent right upper lobectomy for suspicious lung nodule showing: **a** subpleural reticular changes (arrow) and centrilobular ground glass opacities (arrowhead) consistent with interstitial lung abnormality and **b** traction bronchiectasis (arrow) and centrilobular emphysema (asterisk). Non-malignant histologic findings for this patient included emphysema, histologic fibrosis, respiratory bronchiolitis and peribronchiolar metaplasia (not shown). Additionally, pre-surgical CT chest images of a 58-year-old active smoker (30 pack years) who underwent right lower lobe lobectomy for suspicious lung nodule showing a CT pattern of combined pulmonary fibrosis emphysema (CPFE): **c** centrilobular and paraseptal emphysema (arrows) in upper lobes, and **d** reticular changes with honeycombing (arrow) in the right lower lobe. Corresponding histologic findings of this patient are shown in Fig. 4

**Fig. 4:**
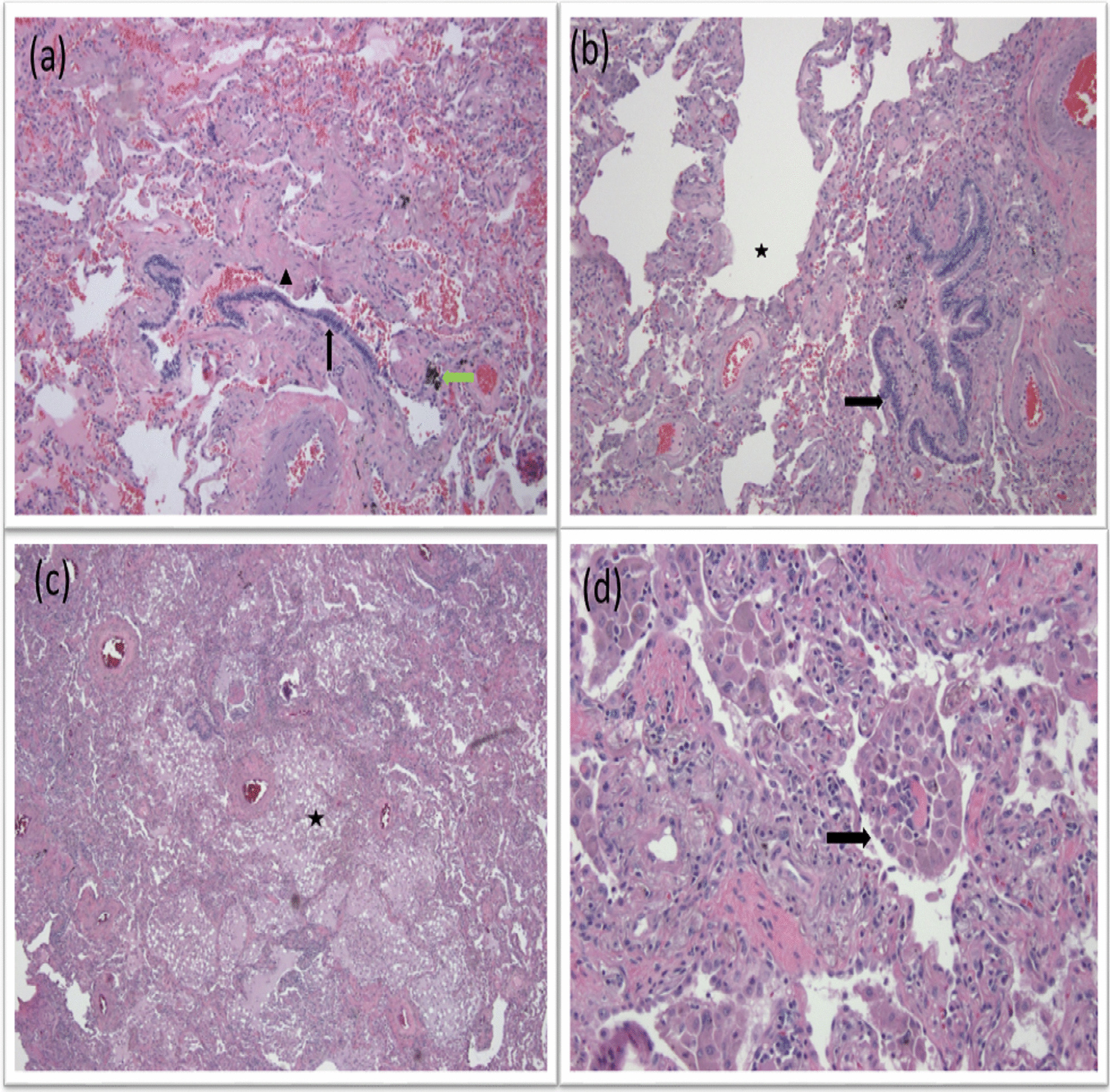
Photomicrographs from 58-year-old active smoker (30 pack years) who had radiographic ILD pattern of combined pulmonary fibrosis emphysema (CPFE) and underwent right lower lobectomy for suspicious nodule showing: **a** peribronchiolar metaplasia (PBM, black arrow), interstitial fibrosis (arrow head), anthracotic pigment deposition (green arrow), and [100×] (**b**) emphysema (asterisk) and PBM (arrow) [100×] (**c**) pattern of desquamative interstitial pneumonitis (DIP) where alveoli were diffusely and extensively filled by macrophages (asterisk) [40×] (**d**) DIP with pigmented macrophages filling the alveoli (arrow) [200×]

Univariate analysis of clinical, radiographic and pathologic variables for ILA/ILD patterns are provided (Fig. 2). Ever smoking status and ≥ 30 pack years of smoking predicted ILA/ILD patterns by two-fold (OR 2.46, 95% CI [1.22–4.96] and OR 2.21, 95% CI [1.44–3.39], respectively). Clinical evidence of COPD as well as pathologic and radiographic findings of emphysema were also associated with presence of ILA/ILD (OR 1.56, 95% CI [1.01–2.40], *p* = 0.044; OR 4.62, 95% CI [2.79–7.66], *p* < 0.001 and OR 3.16, 95% CI [2.04–4.90], *p* < 0.001, respectively). Additionally, radiographic findings of paraseptal emphysema and isolated traction bronchiectasis predicted ILA/ILD. Pathologically, presence of PBM (OR 6.92, 95% CI [2.37–20.23], *p* < 0.001), any pulmonary fibrosis (OR 4.51, 95% CI [2.11–9.65], *p* < 0.001), RB (OR 4.21, 95% CI [1.96–9.05], *p* < 0.001) and anthracosis (OR 1.57, 95% CI [1.01–4.48], *p* = 0.043) were associated with ILA/ILD. Figure 3 provides representative CT chest images of patients with ILA and ILD in the cohort. Figure 4 shows corresponding non-malignant histological features identified on the surgical specimen of an ILD patient.

The associations between specific radiographic ILA and ILD findings with the pathological features were explored using multivariate logistic model (Table 4). Of ILA findings, CL-GGO showed an association with PBM (OR 3.98, 95% CI [1.08–14.60], *p* = 0.037) whereas mixed CL-GGO and subpleural reticular changes were associated with histologic evidence of any pulmonary fibrosis (OR 2.98, 95% CI [1.16–7.63], *p* = 0.022). Subpleural reticular changes did not independently identify pathological predictors. Radiographic ILD patterns demonstrated a strong association with histologic evidence of any pulmonary fibrosis (OR 2.48, 95% CI [1.24–4.98], *p* = 0.010), PBM (OR 2.56, 95% CI 1.05–6.22], *p* = 0.038) and RB (OR 2.14, 95% CI [1.01–4.54], *p* = 0.046).

**Table 4:** Logistic regression analysis showing association between radiographic ILA and ILD patterns and pathologic findings in the cohort (N = 352)

	Model consisted of path findings (any fibrosis, PBM, RB, DIP, OP, anthracosis)	Odds ratio	95% CI	*p* value
Individual radiographic ILA findings (N = 124, 35.2%)				
1. Centrilobular GGO (n = 28)	PBM	3.98	1.08–14.60	0.037
2. Subpleural reticulation (n = 53)	None significant			
3. Mixed 1 + 2 (n = 26)	Any fibrosis	2.98	1.16–7.63	0.022
4. Non-emphysematous cysts (n = 28)	DIP	3.55	0.86–14.57	0.078*
Radiographic ILD patterns only (N = 62, 17.6%)	a. Any fibrosis	2.48	1.24–4.98	0.010
	b. PBM	2.56	1.05–6.22	0.038
	c. RB	2.14	1.01–4.54	0.046

(*) indicates trend towards significance

*DIP* desquamative interstitial pneumonia, *GGO* ground glass opacity, *ILA* interstitial lung abnormalities, *ILD* interstitial lung disease, *OP* organizing pneumonia, *PBM* peribronchiolar metaplasia, *RB* respiratory bronchiolitis, *RB-ILD* respiratory bronchiolitis–interstitial lung disease

Since emphysema was a strong predictor of ILA/ILD, logistic regression analysis evaluating the relationship of histologic emphysema, other path features and smoking was performed (Table 5). These analyses suggested associations of histologic emphysema to any fibrosis (OR 2.24, 95% CI [1.15–4.37], *p* < 0.017), RB (OR 4.11, 95% CI [1.86–9.10], *p* < 0.001), anthracosis (OR 2.93, 95% CI [1.81–4.75], *p* < 0.001) and ≥ 30 pack years of smoking (OR 1.68, 95% CI [1.05–2.68], *p* = 0.038).

**Table 5:** Logistic regression analysis showing association between histologic emphysema (n = 188, 53.4%), other histopathologic features and smoking in the cohort (N = 352)

	Odds ratio	95% CI	*p* value
Any path fibrosis	2.24	1.15–4.37	0.017
RB	4.11	1.86–9.10	< 0.001
Anthracosis	2.93	1.81–4.75	< 0.001
≥ 30 pack years smoking	1.68	1.05–2.68	0.030

*RB* respiratory bronchiolitis

Patients with radiographic ILA/ILD were at increased risk for mortality (odds ratio 4.13, [95% CI of 1.84–9.25]). After adjusting for age, gender, BMI, and primary lung cancer status, multivariate logistic regression showed GERD (OR 2.53, [95% CI 1.25–5.15], *p* = 0.010), ≥ 30 pack year smoking (OR 2.32, [95% CI 1.01 -5.30], *p* = 0.046), radiographic ILA/ILD patterns (OR 4.04, [95% CI 1.74–9.40], *p* = 0.001) and radiographic isolated honeycomb changes (OR 7.32, [95% CI 1.35–39.84], *p* = 0.021) as predictors of mortality in the study cohort (Table 6).

**Table 6:** Logistic regression model showing predictors of mortality in the cohort (N = 352)

Variable	Odds ratio	95% CI	*p* value
GERD	2.53	1.25–5.15	***0.010***
≥ 30 pack year smoking	2.32	1.01–5.30	***0.046***
Radiographic ILA/ILD patterns	4.04	1.74–9.40	***0.001***
Radiographic paraseptal emphysema	0.61	0.27–1.40	0.250
Radiographic isolated honeycombing	7.32	1.35–39.84	***0.021***
Histologic any fibrosis	0.84	0.34–2.03	0.693

Bold italics* p*-value suggest statistical significant* p*-value (alpha < 0.05)

*GERD* gastro-esophageal reflux disease

## Discussion

The study cohort revealed a high prevalence (52.8%) of subclinical ILA and ILD patterns which were significantly associated with ever smoking and intensity of smoking (≥ 30 pack years). While smoking status and smoking intensity have been significantly associated with ILA, our prevalence is greater than previously reported [5, 22]. There’s a lack of standardization of ILAs which makes strict comparisons of this prevalence between investigations difficult. Our study assessed ILA/ILD patterns on visual inspection of pre-surgical resection CT chest while others have relied on quantitative high attenuation areas (HAAs) to define ILAs [22, 23]. Pathological investigation which utilized tissue resected for lung cancer reported a similarly high prevalence of radiographic interstitial changes [13]. Applying different methodology, a comparable rate of interstitial fibrosis (60%) was pathologically identified in smokers [24, 25].

To better explain the paradox of variable clinical and pathophysiologic responses to an identical risk factor (i.e. smoking), the need for studies characterizing interactions between emphysema, pulmonary fibrosis and ILAs have been acknowledged [2]. A clinical diagnosis of COPD, radiographic and histologic emphysema predicted a presence of ILA/ILD in our smoking cohort. Histologically, the observation of any pulmonary fibrosis predicted ILAs (mixed ground glass opacities and subpleural changes) and ILDs in our cohort. Moreover, patients with pathologic evidence of emphysema in the resected lungs were more likely to have associated pulmonary fibrosis, RB, anthracosis and to have smoked for ≥ 30 pack years. Clinically, considerable overlap in physiological and radiographic features in smokers have led to increasing recognition of CPFE [26, 27]. In lung tissue resected with nodules, fibrosis could be detected in 50 to 70% while emphysema was observed in 20% or more [28]. One-third of patients undergoing lung volume reduction surgery for severe emphysema were noted to have unsuspected histologic findings including interstitial and subpleural fibrosis in approximately 20% of patients [29]. Pathological and radiological evidence of a relationship between lung fibrosis and emphysema has previously revealed two patterns [4]. The first pattern reflects a fibrosis which is initially localized and frequently clinically occult (respiratory bronchiolitis (RB), RB with interstitial lung disease (RB-ILD), RB-ILD with fibrosis, airspace enlargement with fibrosis, and smoking-related interstitial fibrosis (SRIF)). Such fibrosis can be an integral part of the emphysematous process and is characterized by increased collagen in the respiratory bronchiole accompanied by CL emphysema [30, 31]. The fibrosis can extend into adjacent alveolar walls (in subpleural and CL regions) in approximately half of current and ex-smokers [32, 33]. The second pattern includes fibrosis of a diffuse interstitial pneumonitis, most commonly UIP, mixed with CL or PS emphysema. These cases are included in CPFE [24, 34–37]. On pathological examination, there can be areas of (1) pure fibrotic interstitial pneumonia, (2) pure emphysema, and (3) fibrosis wrapped around emphysematous spaces with fibrotic changes, most frequently consistent with UIP [26].

Of various histologic features reported in this cohort, PBM was almost seven times more likely to predict radiographic ILA/ILD patterns. More specifically, PBM strongly associated with CL-GGO of ILA patterns and with ILD only pattern on CT chest. PBM is a non-specific reaction to bronchiolar injury, characterized by fibrosis and proliferation of bronchiolar epithelium along peribronchiolar alveolar walls. PBM is a common finding in ILDs ranging from 11% in RB-ILD to 50% in DIP, NSIP, and HP and 59% in UIP cases [38]. PBM identification in known airway centered lung injuries of RB, DIP and chronic HP suggest that PBM could be included in the response to particles including cigarette smoke. Such airway centered parenchymal fibrosis is frequently seen with the respiratory bronchiole of asymptomatic current or ex-smokers [32]. Pathologic-CT correlative investigation which evaluated cigarette smoking-induced parenchymal changes revealed that GGO on HRCT was associated with an accumulation of pigmented macrophages in alveolar spaces associated with fibrosis [39]. In addition, poorly defined parenchymal micronodules demonstrated pathological findings of bronchiolectasis with peribronchiolar fibrosis. Thus, the HRCT abnormalities of respiratory bronchiole (i.e. CL-GGO) and PBM appreciated in our cohort could correspond to some combination of the inflammation and fibrosis around the terminal bronchioles [40, 41]. Non-emphysematous cysts ILA pattern was observed in 8% of cohort and it showed a possible association with histologic DIP. Small (< 2 cm) thin-walled cysts admixed with GGO can be a unique feature in about one-third of DIP patients [42]. Cysts in DIP typically characterize the dilated bronchioles and alveolar ducts secondary to bronchiolar stenosis [43]. Non-emphysematous cysts of ILAs in our cohort may represent as an early response to smoking-related injury with a predilection towards development into DIP.

With availability of pre-surgical PFTs for majority of patients (> 95%), our findings provide comprehensive physiological evaluation and its interaction with diverse clinical, radiographic and pathologic variables recorded in the cohort. Decreased DL_CO_ (< 70% predicted) was 1.5 times likely to predict ILAs/ILD in our cohort with lower mean DL_CO_ values associated with ILA/ILD. As reported among patients with CPFE, the presence of interstitial abnormalities can augment percent predicted FEV_1_ with no difference in % predicted FVC, resulting into the pseudo-normalization of FEV_1_/FVC ratio [44, 45]. Moderate to severely reduced DL_CO_ is a consistent and characteristic finding mentioned in CPFE patients reflecting the additive effects of emphysema and fibrosis on the reduction in surface area available for gas exchange [46]. A greater exposure to cigarette smoke and reduced DL_CO_ were predictors for CPFE in a cohort of IPF patients [16]. Similar deleterious effect of emphysema in patients with interstitial features were reported by Ash SY et al. based on limited availability of DL_CO_ findings in their study participants (< 5%) [10]. Therefore, an isolated reduction in DL_CO_ can be a diagnostic clue for ILAs, particularly in a cohort with a high prevalence of emphysema.

In addition to GERD and ≥ 30 pack years of smoking, radiographic ILA/ILD and isolated honeycombing were the greatest predictors of mortality (OR 4.04 and 7.32, respectively) in our cohort. This was independent of age, BMI, lung cancer finding on resected nodule, and radiographic or histologic emphysema. The presence of ILA with the definite fibrosis pattern (pulmonary parenchymal architectural distortion with traction bronchiectasis and honeycombing) was previously associated with the highest risk of progression and increased mortality on 5-year follow up [47]. Traction bronchiectasis was similarly associated with shorter survival among subjects with ILA [48]. ILAs in a lung cancer screening population of relatively healthy smokers (> 20 py) were also associated with mortality but this was regardless of the interstitial morphological phenotype [49]. Analysis of a large research cohort showed an association of increased all-cause mortality with ILAs with the higher median pack years of smoking seen in subjects with ILAs [50].

We report several limitations for our study. It was a retrospective study at a single tertiary medical center which poses inherent limitations. Assertion of cigarette smoking behavior was obtained retrospectively introducing recall bias. A majority of participants were white. Our qualitative determination of ILAs instead of quantitative assessment may affect the repeatability of our findings; however, others have reported poor positive predictive value of quantitative high attenuation area (HAA) [8]. Moreover, considerable interobserver variability has been known to exist for fibrotic pattern recognition on a CT scan, even among thoracic experts [51, 52]. We did not quantify the extent of pulmonary emphysema, which could have assisted in defining its interplay with lung fibrosis and its impact on important clinical outcomes. Surgical resection was performed for suspected pulmonary nodules/masses but not designed to provide information about non-malignant path findings including ILA. Recent reporting guidelines recommend that pathologists should record and categorize the presence of non-neoplastic lung parenchymal changes including emphysema, RB, and interstitial fibrosis with any discernible patterns [53]. In contrast, strengths of the study include relatively large cohort with consecutively included patients undergoing surgical resection, detailed description of radiologic findings and their correlation with histological features.

## Conclusion

With the highest national smoking rates observed in the Appalachian region, radiographic subclinical ILA/ILD patterns were highly prevalent. Correlating clinical, radiographic and pathologic characteristics, our analysis provided evidence of co-existence of emphysema and ILA/ILD either radiologically or histologically in association with smoking. In addition, the study finds a unique association of PBM to CL-GGO in ILA patterns which may be indicative of smoking associated small-airways injury. While confirming the role of ILA/ILD and honeycombing for increased mortality, GERD and cigarette smoking intensity showed a significant impact on mortality in our cohort. Analogous to CPFE, preserved spirometry and isolated reduction in DL_CO_ might be a key physiological consequence of early interstitial changes with smoking. Additionally, the study participants showed the presence of diverse non-malignant lung pathologies from cigarette smoke exposure which could represent a continuum of injury process at a tissue level. Considering self-limiting nature of some of these inflammatory changes, greater emphasis should be placed on earlier identification of subclinical ILAs in emphysema patients to allow implementation of effective smoking cessation strategy.

## Supplementary Information

**Additional file 1.** Radiographic and pathologic case definitions of emphysema, ILA and ILD.

## Data Availability

All data generated or analyzed during this study are included in this published article.

## Abbreviations

CPFE: Combined pulmonary fibrosis and emphysema
CL: Centrilobular
DL_CO_: Diffusion capacity for carbon monoxide
DIP: Desquamative interstitial pneumonia
FEV_1_: Forced expiratory volume in one second
FVC: Forced vital
GERD: Gastro-esophageal acid reflex
GGO: Ground glass opacity
ILAs: Interstitial lung abnormalities
ILDs: Interstitial lung diseases
IPF: Idiopathic pulmonary fibrosis
NSIP: Non-specific interstitial pneumonia
OR: Odds ratio
PBM: Peribronchiolar metaplasia
PFT: Pulmonary function test
PLCH: Pulmonary Langerhans cell histiocytosis
RB-ILD: Respiratory bronchiolitis–interstitial lung disease
RV: Residual volume
SRIF: Smoking-related interstitial fibrosis
TLC: Total lung capacity
UIP: Usual interstitial pneumonia
