# Supplementary material for: Interstitial lung abnormalities and interstitial lung diseases associated with cigarette smoking in a rural cohort undergoing surgical resection
Source: BMC Pulm Med. 2022 Apr 29;22:172. doi: 10.1186/s12890-022-01961-9 (PMC9055776; doi:10.1186/s12890-022-01961-9)
Supplement: Supplementary file 1 — Additional file 1. Radiographic and pathologic case definitions of emphysema, ILA and ILD. [file 12890_2022_1961_MOESM1_ESM.docx]

**Supplementary material for manuscript titled “Interstitial Lung Abnormalities and Interstitial Lung Diseases Associated with Cigarette Smoking in a Rural Cohort Undergoing Surgical Resection”**

**Case definitions for radiographic evaluation:**

1. Interstitial lung abnormalities (ILAs):

CT scan of chest (slice thickness of 1 mm and 3 mm for lung and mediastinal windows, respectively) were characterized in accordance to previous literature to detect incidental non-dependent abnormalities, including diffuse centrilobular ground glass opacities (GGO), subpleural reticular changes, architectural distortion, traction bronchiectasis, honeycombing, and non-emphysematous cysts involving at least 5% of a lung zone.^1-3^

We excluded imaging findings if they were restricted to dependent lung zones, focal paraspinal fibrosis, focal or unilateral abnormality, interstitial pulmonary edema or aspiration related findings of tree-in-bud or patchy ground glass and definite ILD patterns.

1. Interstitial lung disease (ILD) patterns:

Various radiographic ILD patterns were recorded including usual interstitial pneumonia (UIP), probable UIP, non-specific interstitial pneumonia (NSIP), respiratory bronchiolitis-ILD (RB-ILD), pulmonary Langerhans cell histiocytosis (PLCH), desquamative interstitial pneumonia (DIP), combined pulmonary fibrosis and emphysema (CPFE), organizing pneumonia (OP) and unclassifiable patterns.^4-9^

1. UIP: UIP is the hallmark radiologic pattern of idiopathic pulmonary fibrosis (IPF). Honeycombing is a distinguishing feature of UIP which can be seen with or without peripheral traction bronchiectasis or bronchiolectasis. Subpleural and basal predominant reticular changes seen frequently, however, distribution can be heterogeneous and asymmetrical in 25% of cases. Non-dominant mild GGO can be seen along with reticular changes.
2. Probable UIP: this pattern consists of subpleural, basal-predominant reticular changes with peripheral traction bronchiectasis or bronchiolectasis but usually lacks honeycombing. As with UIP, it may be associated with non-dominant mild GGO.
3. NSIP: it is characterized by homogeneous lung involvement without an obvious apicobasal gradient, presence of extensive GGO, a finer reticular pattern, and micronodules. There can be an immediate subpleural sparing which is a relatively specific sign for NSIP. Traction bronchiectasis is visible in advanced disease of fibrotic NSIP.
4. RB-ILD: this smoking associated ILD pattern is characterized by centrilobular nodules in combination with ground-glass opacities and bronchial wall thickening. Distribution of these findings is usually upper lobe predominant. Coexisting moderate centrilobular emphysema is common.
5. P-LCH: this smoking associated ILD is usually distinguished by relative sparing of lung bases, particularly costophrenic angles in its distribution. In the early stages, centrilobular nodules with or without cavitation are observed, whereas in late stages, there are bizarre shaped thin wall cysts. Associated findings including GGO, reticular opacities, mosaic attenuation, and emphysema.
6. DIP: this smoking associated ILD is characterized by diffuse GGO with a peripheral and lower lung lobe predominance. Additional frequent CT findings include spatially limited irregular linear opacities and small cystic spaces, which are indicative of fibrotic changes. Imaging findings of RB-ILD and DIP may often overlap and can be indistinguishable from each other.
7. CPFE: it is described by presence of centrilobular and/or paraseptal emphysema in the upper lobes and diffuse interstitial features suggestive of pulmonary fibrosis (reticular opacities with peripheral and basal predominance, honeycombing, architectural distortion and/or traction bronchiectasis or bronchiolectasis) in the lower lobes. Focal non-prominent GGO can be an associated finding.
8. OP: CT lung abnormalities of OP show a characteristic lower lobes predominant airspace opacity (either GGO or consolidation) in peripheral or peribronchial distribution. Occasionally, the outermost subpleural area is spared. The air bronchograms and mild cylindrical bronchial dilatation are seen in the consolidated lung zones.
9. Emphysema:

Visual assessment of emphysematous region on CT chest was performed to determine the dominant types of emphysema.^10,11^

1. Centrilobular emphysema: this is the most common type of smoking related emphysema and seen usually in upper lung zones. It is characterized by well or poorly defined areas of low attenuation surrounded by normal lung. Centrilobular pulmonary arteries or arterioles can be seen into the center of each secondary pulmonary lobule.
2. Paraseptal emphysema: this type of emphysema is characterized by subpleural and peribronchovascular foci of low attenuation separated by thickened interlobular septa which usually represents associated mild fibrosis. It is often observed along with mediastinal and peripheral pleura with predilection for middle and upper lungs. Rows of paraseptal emphysema can mimic honeycombing however, cysts in paraseptal emphysema are larger and there’s lack of associated architectural distortion and other signs of fibrosis.
3. Bullous emphysema: it is described as low attenuation areas >1 cm in diameter with a thin wall. It can be found in all types of emphysema but are most seen with paraseptal type and often located at the upper lung zones.
4. Panacinar emphysema: it refers to the diffuse emphysematous destruction of the secondary pulmonary lobule. Considering the uneven distribution of the disease, the low attenuation region on CT chest is not evenly distributed within the lobule. In localized form of panacinar emphysema, it has a polygonal border representing interlobular septa and perilobular large vessels seen at the border of the lesion. In diffuse form, the margin of emphysema becomes ill-defined. In smokers, centrilobular emphysema can be seen in association with panacinar emphysema. With alpha-1 antitrypsin deficiency, panacinar emphysema often shows lower lobe predominance.

**Case definitions for pathologic evaluation**

The following definitions were utilized to establish consensus of histopathologic features.^6,12,13^

1. Emphysema, which was almost typically of the proximal acinar (centrilobular) or distal acinar (paraseptal) type, was defined as airspace dilation in at least two low-power fields with or without associated respiratory bronchiolitis.
2. Respiratory bronchiolitis was noted when increased numbers of alveolar macrophages seen in respiratory bronchioles and immediately adjacent alveoli as well as evidence of mild fibrosis and/or smooth muscle hypertrophy.
3. Peribronchiolar metaplasia (PBM) was noted when bronchiolar epithelialization of alveolar spaces was present, specifically in a bronchiolocentric injury pattern.
4. Fibroblastic foci were defined as rounded intraalveolar collections of fibroblasts and collagenous matrix.
5. When more extensive and associated with chronic inflammation (Masson bodies) and architectural changes, the findings were classified as organizing pneumonia (OP).
6. The presence of interstitial pneumonia was also evaluated, and the pattern was classified if possible.
   1. Cellular NSIP was defined as uniform mild thickening of the alveolar septa containing a monotonous chronic inflammatory infiltrate.
   2. DIP was recognized when plugging and distention of airspaces by numerous alveolar macrophages was present with only minimal thickening of the alveolar septa and absence of fibroblastic foci.
7. Additional features evaluated for each case included anthracosis, focal fibrosis (nonspecific pattern), subpleural fibrosis, architectural distortion, honeycomb changes, granulomatous changes (necrotizing, non-necrotizing-sarcoid like, loosely formed-hypersensitivity pneumonitis (HP) like, calcified) and miscellaneous changes.

**References**:

1. Hatabu H, Hunninghake GM, Richeldi L, et al. Interstitial lung abnormalities detected incidentally on CT: a Position Paper from the Fleischner Society. *Lancet Respir Med*. Jul 2020;8(7):726-737. doi:10.1016/S2213-2600(20)30168-5

2. Washko GR, Hunninghake GM, Fernandez IE, et al. Lung volumes and emphysema in smokers with interstitial lung abnormalities. *N Engl J Med*. Mar 10 2011;364(10):897-906. doi:10.1056/NEJMoa1007285

3. Hata A, Schiebler ML, Lynch DA, Hatabu H. Interstitial Lung Abnormalities: State of the Art. *Radiology*. Oct 2021;301(1):19-34. doi:10.1148/radiol.2021204367

4. American Thoracic S, European Respiratory S. American Thoracic Society/European Respiratory Society International Multidisciplinary Consensus Classification of the Idiopathic Interstitial Pneumonias. This joint statement of the American Thoracic Society (ATS), and the European Respiratory Society (ERS) was adopted by the ATS board of directors, June 2001 and by the ERS Executive Committee, June 2001. *Am J Respir Crit Care Med*. Jan 15 2002;165(2):277-304. doi:10.1164/ajrccm.165.2.ats01

5. Travis WD, Costabel U, Hansell DM, et al. An official American Thoracic Society/European Respiratory Society statement: Update of the international multidisciplinary classification of the idiopathic interstitial pneumonias. *Am J Respir Crit Care Med*. Sep 15 2013;188(6):733-48. doi:10.1164/rccm.201308-1483ST

6. Margaritopoulos GA, Vasarmidi E, Jacob J, Wells AU, Antoniou KM. Smoking and interstitial lung diseases. *Eur Respir Rev*. Sep 2015;24(137):428-35. doi:10.1183/16000617.0050-2015

7. Mueller-Mang C, Grosse C, Schmid K, Stiebellehner L, Bankier AA. What every radiologist should know about idiopathic interstitial pneumonias. *Radiographics*. May-Jun 2007;27(3):595-615. doi:10.1148/rg.273065130

8. Raghu G, Remy-Jardin M, Myers JL, et al. Diagnosis of Idiopathic Pulmonary Fibrosis. An Official ATS/ERS/JRS/ALAT Clinical Practice Guideline. *Am J Respir Crit Care Med*. Sep 1 2018;198(5):e44-e68. doi:10.1164/rccm.201807-1255ST

9. Cottin V, Nunes H, Brillet PY, et al. Combined pulmonary fibrosis and emphysema: a distinct underrecognised entity. *Eur Respir J*. Oct 2005;26(4):586-93. doi:10.1183/09031936.05.00021005

10. Lynch DA, Austin JH, Hogg JC, et al. CT-Definable Subtypes of Chronic Obstructive Pulmonary Disease: A Statement of the Fleischner Society. *Radiology*. Oct 2015;277(1):192-205. doi:10.1148/radiol.2015141579

11. Takahashi M, Fukuoka J, Nitta N, et al. Imaging of pulmonary emphysema: a pictorial review. *Int J Chron Obstruct Pulmon Dis*. 2008;3(2):193-204. doi:10.2147/copd.s2639

12. Berg K, Wright JL. The Pathology of Chronic Obstructive Pulmonary Disease: Progress in the 20th and 21st Centuries. *Arch Pathol Lab Med*. Dec 2016;140(12):1423-1428. doi:10.5858/arpa.2015-0455-RS

13. Kadoch MA, Cham MD, Beasley MB, et al. Idiopathic interstitial pneumonias: a radiology-pathology correlation based on the revised 2013 American Thoracic Society-European Respiratory Society classification system. *Curr Probl Diagn Radiol*. Jan-Feb 2015;44(1):15-25. doi:10.1067/j.cpradiol.2014.07.005
